# Supplementary material for: Social Health Insurance and Healthcare Seeking Behavior in Urban Ethiopia
Source: Ann Glob Health. 2023 Nov 28;89(1):84. doi: 10.5334/aogh.4240 (PMC10691283; doi:10.5334/aogh.4240)
Supplement: Appendix Tables. — Tables A1 to A5. [file agh-89-1-4240-s1.pdf]

Appendix Tables

Table A1
Potential SHI members based on a 2013 labor force survey and estimates from Ethiopian Health Insurance Agency

Sector	Number of employees (Share of total in %)	Sample distribution-*Planned* (Share of total in %)	Sample distribution-*Achieved* (Share in total in %)
Government worker/civil servant [a]	1,410,572	690	1280
	(32.8)	(32.8)	(46.6)
Public sector enterprise [b]	424,039	208	195
	(9.9)	(9.9)	(7.1)
Private sector enterprise [b]	1,789,963	872	949
	(41.7)	(41.5)	(34.5)
NGO workers [b]	84,808	42	92
	(1.98)	(2.00)	(3.4)
Civil servant/public sector	590,024	289	233
enterprise pensioners [a]	(13.75)	(13.76)	(8.5)
Total	4,290,406	2,101	2,749

Notes: [a] From EHIA, actual number of employees in May 2016; [b] Estimated from Central Statistical Office, labor force survey

Table A2
Distribution of sample by sector and city
Planned versus Achieved

	Addis Ababa	Bahir Dar	Hawassa	Mekelle	Sample distribution (Share of total in %)
Government worker/civil servant [a]	372<br>[644]	84<br>[169]	108<br>[202]	126<br>[265]	690<br>(32.8)<br>[1280]<br>[46.6]
Public sector enterprise [b]	112<br>[141]	25<br>[5]	33<br>[48]	38<br>[1]	208<br>(9.9)<br>[195]<br>[7.1]
Private sector enterprise [b]	470<br>[557]	106<br>[60]	137<br>[144]	159<br>[188]	872<br>(41.5)<br>[949]<br>[34.5]
NGO workers [b]	22<br>[42]	5<br>[14]	7<br>[17]	8<br>[19]	42<br>(2.00)<br>[92]<br>[3.4]
Civil servant/public sector enterprise pensioners [a]	155<br>[156]	35<br>[41]	45<br>[1]	53<br>[35]	288<br>(13.76)<br>[233]<br>[8.5]
Total<br>(Share of total in %)	1,131<br>(53.9)<br>[1540]<br>[56.0]	255<br>(12.1)<br>[289]<br>[10.5]	330<br>(15.7)<br>[412]<br>[15.0]	384<br>(18.2)<br>[508]<br>[18.5]	2,100<br>(100)<br>[2749]<br>[100]

Note: Figures for the actual/achieved sample sizes are provided in square brackets.

Table A3
Distribution of civil servants in Addis Ababa

	Names of organization	Total employees	Sample size
Addis Ababa City Administration	Addis Ababa Road Authority	2543	36
	Disaster Preparedness and Control Authority	813	12
	Bureau of Culture and Tourism	518	7
	Bureau of Women and Children	470	7
	Mass Media Agency	374	5
Oromia Regional Administration (based in Addis Ababa)	Auditor General	298	4
	Bureau of Agriculture	332	5
	Water, Mineral and Energy Bureau	288	4
	Bureau of Labor & Social Affairs	300	4
	Bureau of Health	266	4
Federal	Ministry of Agriculture	1401	20
	Ministry of Health	1084	15
	Ministry of Water and Energy	990	14
	Ministry of Education	830	12
	Ministry of Urban Development and Construction	563	8
	Ministry of Finance and Economic Development	804	11
	Ministry of Foreign Affairs	866	12
	EARI (EARO)	3645	52
	Ethiopian Road Authority	1396	20
	Governmental House Agency	1685	24
	Paulos hospital	1479	21
	Addis Ababa University	3324	47
	Ethiopian Radio and Television Agency	2011	28
Total		26280	372

Table A4
Addis Ababa pension paying centers

Sub-city	Selected payment center	Sample Size
Yeka	Woreda 8<br>Tesfa Birhan secondary school	31
Gulele	Post office around Paulos hospital close to CBE branch<br>Addis credit and saving in the premises of Woreda 9 office Rufael	31
Bole	Bole Mikael post office (office in a condominium close to Bole Ring Road Square)<br>Gerji post office Sunshine condominium close to Roba bakery	31
Akaki	Saris – Addis Sefer in the premises of Woreda 8	31
Nefas Silk Lafto	Post office in Gotera condominium	31
Total		155

Table A5
Probability of seeking outpatient care and choice of provider among formal sector employees (marginal effects)

	Seeking treatment	Seeking formal treatment	Choice of formal healthcare provider			
			Health worker Public sector	Health worker Private sector	Doctor Public sector	Doctor Private sector
Sex (female as reference)	-0.047	-0.043	0.006	0.012	0.029	-0.048
	(0.116)	(0.235)	(0.884)	(0.759)	(0.588)	(0.388)
Age (18–34 years old as reference)						
35–55 years old	0.018	0.030	0.032	-0.064	0.062	-0.030
	(0.627)	(0.486)	(0.504)	(0.143)	(0.271)	(0.618)
55 years and older	-0.063	0.030	0.026	-0.116	0.056	0.034
	(0.449)	(0.655)	(0.802)	(0.033)	(0.584)	(0.781)
Household size (1person as ref.)						
2–3 persons	0.093	0.042	0.017	0.133	-0.067	-0.083
	(0.053)	(0.241)	(0.783)	(0.003)	(0.346)	(0.302)
4–5 persons	0.123	-0.048	0.030	0.036	0.070	-0.135
	(0.022)	(0.375)	(0.652)	(0.333)	(0.365)	(0.095)
6 and more persons	0.161	0.001	-0.030	0.082	0.018	-0.070
	(0.001)	(0.991)	(0.699)	(0.135)	(0.833)	(0.480)
Education (less than secondary edu. as ref.)						
Secondary education	-0.092	-0.013	0.037	-0.071	-0.020	0.055
	(0.105)	(0.770)	(0.666)	(0.473)	(0.838)	(0.595)
Tertiary/university education	0.022	-0.018	-0.063	-0.064	-0.106	0.232
	(0.639)	(0.644)	(0.426)	(0.570)	(0.256)	(0.024)
Income (First quintile as reference)						
Second quintile	0.029	0.037	0.025	0.043	-0.136	0.068
	(0.542)	(0.584)	(0.750)	(0.547)	(0.117)	(0.494)
Third quintile	0.016	0.028	-0.045	-0.006	0.116	-0.065
	(0.748)	(0.646)	(0.536)	(0.923)	(0.253)	(0.520)
Fourth quintile	0.014	0.066	-0.021	0.023	-0.096	0.095
	(0.768)	(0.256)	(0.776)	(0.727)	(0.296)	(0.356)
Fifth quintile	0.040	0.056	-0.052	-0.019	-0.212	0.282
	(0.469)	(0.322)	(0.487)	(0.795)	(0.021)	(0.009)
Any household member has HI	-0.018	0.002	-0.037	0.065	-0.039	0.012
	(0.564)	(0.961)	(0.422)	(0.146)	(0.488)	(0.855)
Employment Sector (Public sector as ref.)						
Private/NGO sector	0.000	0.104	-0.037	0.030	0.081	-0.074
	(0.998)	(0.001)	(0.445)	(0.500)	(0.167)	(0.213)
Pensioners	-0.004	0.072	-0.044	-0.000	-0.024	0.067
	(0.947)	(0.098)	(0.589)	(0.998)	(0.772)	(0.579)
N	389	269	308			

Notes**:** The first two columns are based on logit models and the last four columns on a multinominal logit model. All models include control variables for region (city). P-values are in parentheses.
